# Behind the scenes with genomics researchers

**DOI:** 10.3389/fgene.2024.1467404

**Published:** 2024-12-12

**Authors:** Renata Mont’Alverne, Lori Bradford, Cheryl Buckmaster, Graham Strickert, Jason MacLean, Diane Dupont

**Affiliations:** ^1^ School of Professional Development, College of Engineering, University of Saskatchewan, Saskatoon, SK, Canada; ^2^ School of Environment and Sustainability, University of Saskatchewan, Saskatoon, SK, Canada; ^3^ Department of Economics, Faculty of Social Sciences, Brock University, St. Catharines, ON, Canada

**Keywords:** philosophy of genomic research, ethics, legal studies, social science, oil sands process water (OPSW)

## Abstract

Although lab-coat genomics scientists are highly skilled and involved in pioneering work, few studies have examined their perceptions on what they do, and how they relate with others in interdisciplinary work. Recognizing that gap, we were curious to talk with scientists about their current work and positionalities related to the use of genomics for bioremediation. Using unstructured open-ended interviews and thematic analysis, we interviewed researchers with diverse genomics-related expertise. Emerging topics were grouped into two broad categories akin to Bronfenbrenner’s nested developmental model: microsystem matters, comprising technical advances, barriers, and localized concerns; and macrosystem matters, exploring wider reflections and the philosophies of genomics and society. At the microsystem level, findings revealed differences of opinion about methodological steps, but there was agreement about the incompleteness of databases and the absence of established reference values. These two problems may not only impact a project’s progress but also the ability to gauge success, affecting budgeting, human resource needs, and overall stress. At the macrosystem level, scientists voiced concerns about how different social groups perceive and accept genomics applications, as those tend to be viewed by lay persons as genetic interventions. Another focus was on how academic publication slows progress because it is orientated toward positive results while gaps in knowledge could be filled by publishing negative results or methodological barriers. This study underscores scientists’ self-awareness within the genomics discipline, acknowledging how their beliefs and biases shape research outcomes. It illuminates critical reflections essential for navigating societal and scientific landscapes in genomics research.

## Introduction

Advances in genomic sciences led to breakthroughs in environmental health monitoring and cleanup. Examples of successes include the adoption of a genomics toolkit to assess freshwater fish health status in face of environmental stressors (Semeniuk et al., 2022); city-scaled molecular profiles of microorganisms through eDNA to build early detection systems for epidemics (Shamarina et al., 2017); engineered microbes for degrading plastics (Kumari and Chaudhary, 2020); and improvement of pest management through the use of omics databases (Klein et al., 2019). These advances have received much attention in the scientific community, yet scientists behind this work are rarely asked what they think about what they do, and the impacts of their research. Although studies on perceptions of genomics indicate that the public holds polarized views of genomics research and its application (Wirz et al., 2020), few studies have examined how the scientists behind this research conceive of legal, social, ethical, and other aspects of their work in specific applied contexts. In the past, these social science insights were studied separately or as addendums to larger natural science-focused research. Some have characterized genomics scientists as highly skilled and innovative individuals (Huang and Ertug, 2014; Chow-White et al., 2017). However, genomics scientists are also characterized in some contexts in the literature as non-empathetic, patronizing, manipulative in their use of rhetoric with the public, and guilty of stereotyping the public as both ignorant and intolerant of risk (Cook et al., 2004; Simis et al., 2016).

An interesting setting in which to study critical questions at the intersection of genomics and society is in Genome Canada projects, as the project teams embed social scientists in the group (Genome Canada, 2022). For the past year, we have been part of a team addressing gaps in research on genomically-informed constructed treatment wetlands as a potential treatment within a suite of options for oil sands process affected water (OSPW). As social scientists with different backgrounds (psychology, water-security, environmental connectivity, economics, and law), we were curious about the perceptions scientists on the team had of themselves, the research team, and their current work on this large-scale interdisciplinary genomics and bioremediation project. To provide an opportunity to share the scientists’ voices and to enhance public understanding of genomics and its ethical, environmental, economic, legal, and social (GE^3^LS) dimensions within one large-scaled applied genomics project, we examined a selection of thoughts and beliefs as a starting point for deeper examination of emergent themes on how genomics scientists think about their work. Our goal was to understand their views of their work through an interdisciplinary lens and through research-creation, defined as “an approach to research that combines creative and conventional research practices, and supports the development of knowledge and innovation through artistic expression, scholarly investigation, and experimentation” (SSHRC, 2020). We aimed to ground the existing spectrum of characterizations of genomics scientists in the literature with these scientists’ expectations and ideas about their work in a bioremediation research project. This examination was not part of the original project funding application. It developed naturally during virtual visits, which were meant for the social science team to meet the genomics scientists. Insights from these conversations sparked a desire for broader sharing of information.

## An exploratory case study, ethnography, and artistic research-creation

We employed an exploratory case study approach (Yin, 2003; Baškarada, 2014) that integrates ethnography of science (Tweney, 2004) with thematic analysis (Rapley, 2001; Ivey, 2023) and grounded theory (Heydarian, 2016). The theoretical foundation of this method hinges on combining these approaches to deepen our understanding of the interactions between scientific practices and their broader contexts. Ethnography provided a detailed, qualitative exploration of the social dimensions of scientific work in genomics laboratories and spaces in one project. Virtual visits consisting of open-ended interviews and tours of the lab were used to capture rich, context-specific data. Thematic analysis of transcripts and ethnographic notes (Emerson et al., 2011), by at least two members of the social science team using an inductive coding process (Gibbs, 2007), helped to identify patterns across these data. An example of a pattern is freely raised concerns around the health and wellbeing of staff and students in the labs. This was coded to a microsystem level, since it was focused on individual health and what members of the local lab could do to enhance it during COVID years. The combined case study, ethnographic, and grounded approach allowed us to generate insights from the coded data, without imposing preconceived notions, for example, in the previous instance, we did not go into coding looking for “health impacts at the local level”. Yet, it emerged freely as a concern from interviews. Together, these methods facilitated iterative refinement of emerging themes, which were then framed within Bronfenbrenner’s ecological systems model (1977) due to the locating of thematic patterns within local and global systems, allowing us to contextualize individual laboratory practices within broader societal and scientific pressures and systems.

Regular progress meetings ensured verification of the analyses through a collaborative and reflexive process. The social science team shared themes and exemplars with the whole project team and these were adapted and verified until consensus or majority agreement was reached. The inclusion of a visual artist throughout all stages of the research-creation process enriched the ethnographic and thematic insights, generating visual interpretations of the findings, which also contributed to knowledge creation (Sweet et al., 2020; SSHRC, 2020).

**FIGURE 1:**
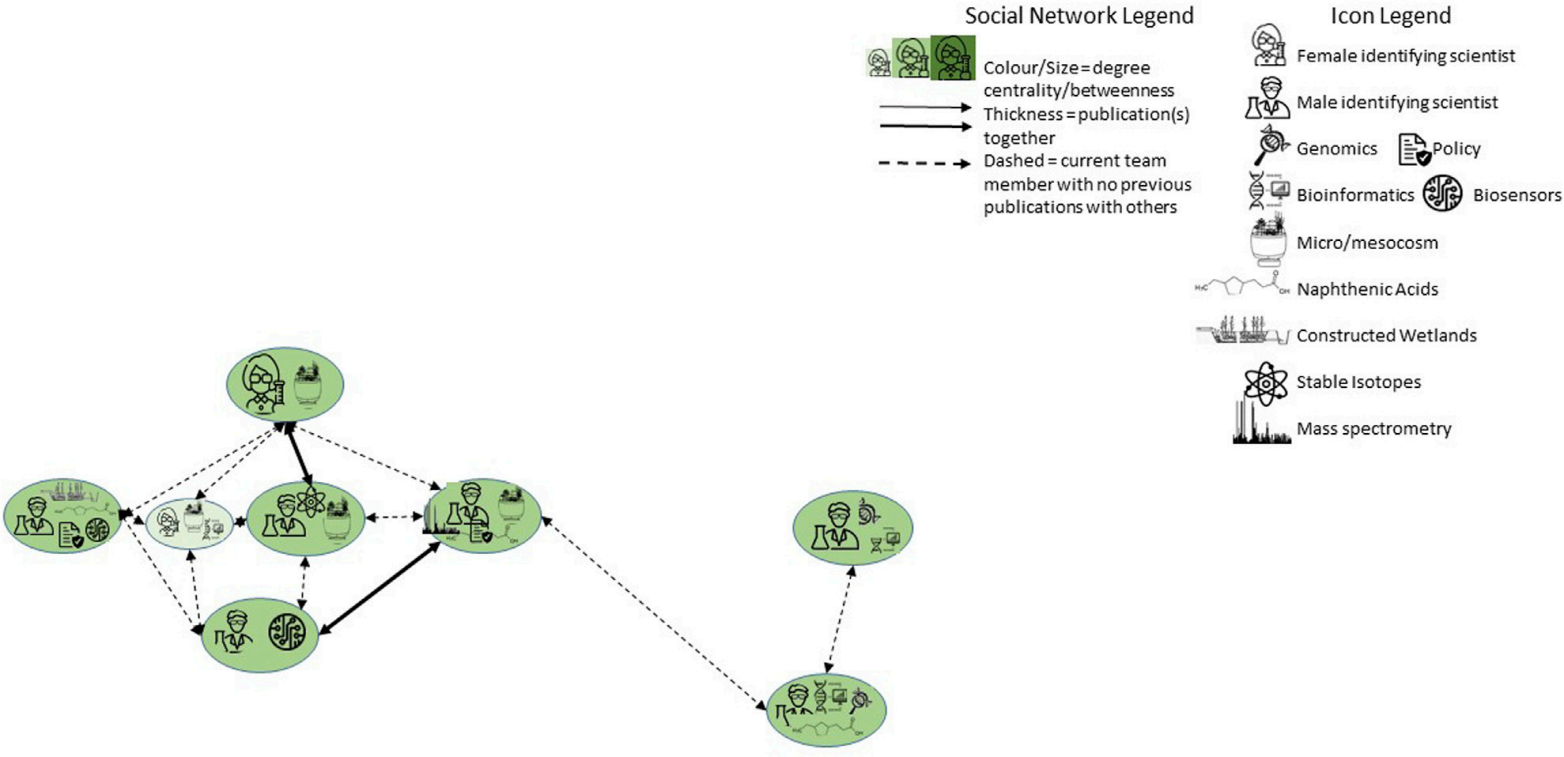
Social network map of participants in virtual visits.

Between October 2021 and April 2022, eight researchers in the project agreed to have either themselves (as principal investigators) or a trusted collaborator (e.g., a laboratory manager or postdoctoral fellow) interviewed for 60–90 min, by at least two members of the social science team and the visual artist. Participants included two individuals who identified as female and six as male, all with a variety of genomics-related expertise. Their previous experiences with each other ranged from no former collaboration to having co-authored peer-reviewed publications (Figure 1). Conversations followed an unstructured format, being guided by two loose questions:

1. What are some methods you are working on or have developed over the last year that will help the overall Genome Canada project?
2. What are some questions about genomics that you and your research team are grappling with right now (can be experimental, moral, ethical, social, legal, scientific, etc.)?

Participants identified their specific fields or methodological expertise, and the focus of their laboratory work within the program. When possible through Zoom, they conducted a walking tour of their facilities with a portable device so they could demonstrate and explain equipment used for experiments and analyses.

## Results and discussion

**FIGURE 2:**
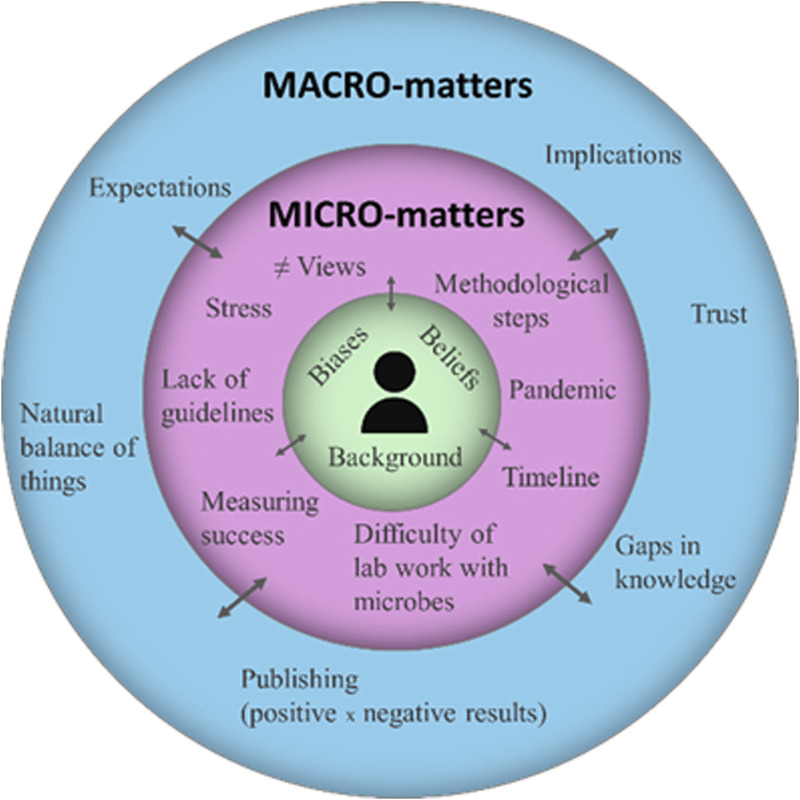
A visualization of topics of interest for genomics’ researchers involved in this case study, based on Bronfenbrenner’s development model.

Different topics emerged during the interviews and, although they were thematically connected to the overall program, they were grouped into two broad categories reflecting the nested developmental model of Bronfenbrenner (1977): microsystem matters, comprising technical advances, barriers, and concerns at the individual and local level; and macrosystem matters, exploring wider reflections and the philosophies of genomics and society (Figure 2).

### Microsystem matters

Interviews began with participants explaining the activities performed in their laboratory. They described novel methods, including the mesocosm system developed specifically for the Genome Canada project, selected mass spectrometry analyses, and the tracing of the fate of toxins within specified plants and animals of cultural and scientific value in constructed wetlands. As explained by one interviewee, the mesocosms were important first steps to establishing plant and microbe community effectiveness at processing toxins from oil sands water by providing throughput samples for further analysis by other laboratory scientists and bioinformaticians. The interviewee stated:

… so these tanks, that [researcher] also uses, many researchers have not used this method to determine naphthenic acids (NA) degradation or even toxin degradation. They actually use multiple tanks, so it doesn’t actually stay in one system. So, this is a new method that I think it was designed for this project, using a single reservoir and having surface flow water to test for degradation and like develop these greenhouse wetlands (INT-6).

Of note was also the internal debate on which approach to pursue, as researchers may have a specific preferred way of doing things. An area where this became clear for us was in the use of biostimulation (modification of the environment to stimulate existing organisms) or bioaugmentation (microbial inoculation in the environment). Some participants agreed with the use of biostimulation – “if you have more plants, then you’re going to stimulate more the microbes and then the microbes are probably going to be more efficient at degrading the contaminants” (INT-1); others expressed excitement mixed with doubt for bioaugmentation – “we’ve talked about it a lot, and I think that augmentation does sound very nice that you can enrich for microbes and then put them into the plant. … there are some examples in the literature where people claim that augmentation works” (INT-4); and some indicated confidence, with the work already in progress – “I have an undergraduate student now, we’ve taken these cultures and we’re bioaugmenting them with different bacteria to see if that will actually help” (INT-7). However, we also could sense unease, expressed by one participant, as follows: “I guess I have concerns, but when it comes to situations like this, you are kind of trading off one for the other. And if it takes some biostimulation or bioaugmentation to treat that water, then we may solve one problem, we may create another, but what does the risk benefit of both of those scenarios look like?” (INT-8).

When discussing their perceptions of how samples are processed and analyzed, participants also stated:

how we analyze, say, samples for the Genome Canada project versus, say, some other projects such as [project names] doesn’t really matter to the instrument and to how we process samples in the lab: there is a lot of very common steps (INT-5).

They explained that “we are not reinventing the wheel; at some point we are just making use of available techniques” (INT-3) and asserted that “it is just applying existing methods to do what you want to do” (INT-1), adding that “the interpretation of the data will of course depend on the types of samples and the questions that are being asked” (INT-5). A common observation was the difficulty of working with microbes, associated with complexities in isolating environmental microbes, cultivating and growing them in a laboratory, and identification, as some environments have been poorly characterized, and most databases are incomplete:

There is a database of microbes, but … it is incomplete. If you typically get DNA out of a sample, let’s say soil or sediment, typically half of that DNA has no [matching] data, it is just unknown. Some people call it dark matter or something, it is just that the DNA exists but we don’t know what it is; it is not present in the database that we use. And the problem is that to do that, to be able to identify the DNA we extract, we need to first isolate microbes and then sequence their genome and have this genome in a database. And, like I said, it is very difficult to get microbes out of their environment, so most of the microbes are not cultivated yet. I would say probably 95% of microbes are not cultivated and sequenced yet and, the ones that are, are mostly from humans and their pathogens and things like that. Environmental microbes you have databases that are very incomplete, which makes our task a bit more difficult… (INT-1).

If we try to take an environmental sample and grow it in the lab, it turns out we can only culture about 1% of what is actually out there. Like, at best. So, a lot of what we know about microbiology and those sequences, those are only DNA-based, nobody knows what they do, they don’t know what their physiology is, yeah, so that is a limitation in this field that I would say that in the field of microbial ecology this is a big question. For me, I like to see what they can do rather than who they are, I’ve always been more of that mindset, but there are people that do a lot of what is their potential, who is there rather than better understanding what they are actually doing in the real world. So, I think kind of marrying those things together is always a challenge (INT-7).

The conversation about the difficulties of lab work naturally led to participants describing challenges they faced because of closures and equipment failure over the years of the SARS-CoV-2 pandemic. Participants described difficulties with procuring supplies; maintaining functionality and accuracy of equipment due to less usage; obtaining results; and keeping up with original estimated timelines, which could delay students in finishing their graduate programs; and keeping personnel employed through applying for grant extensions and extra pandemic-related supports. While some issues have been ongoing concerns for laboratory technicians and scientists (Carey, 2018; Kowalczyk, 2015; Lewis and Gospel, 2015), the participants noted that the pandemic aggravated these scenarios, and participants’ concerns echoed other scientists’ globally (Omary et al., 2020). One participant describes the frustration:

Things have been slow in the lab; we have only just, I would say weeks to months ago we opened, and we are still struggling to find our feet. A lot of the machines were down for at least a year and a half, and they don’t like being turned off for that long: some of the seals get brittle, we get leaks, the electronics don’t want to turn back on… So, we have three main instruments in the lab and it has taken us the best part of six months to get two of them kind of running again, but we are still not where we want to be. And we are not alone. A lot of other labs throughout Canada, and throughout the world, are struggling to get back on their feet. So, my concern, if it is a concern, is [that] whatever time timetable we had at the start of the project needs to be revisited. … if we continue to have some of the progress, now like, although it is limited, the restricted access to lab, we are still kind of operational but not anywhere near where I would like it to be, so that is probably going to impact productivity. So, I would like to lower expectations. We are about a year behind already. Things could change, and a lot has to do with lab closures and equipment failure. And that is not specific to [the] Genome Canada project (INT-5).

Participants reported that steps in one program activity led to subsequent activities in laboratories across the country, such that delays at any point affected the whole program. For example, the delay in securing key pollutant concentrations after the first experiment impacted the mesocosm timeline, as results were needed to adjust the variables at the next stage of experiments. The interview participants were aware of the sequence of steps in the design of interdisciplinary work and how other labs and the GE^3^LS team relied on their results to progress. Participants demonstrated a sensitivity to colleagues and awareness of the need to communicate across the project team. Nevertheless, they pointed out that these delays also allowed the team to look at things differently—to do “slow science” (Stengers, 2018), understand how the pieces fit together, and learn things that they otherwise would not have learned.

Lastly, researchers commented on the guidelines for naphthenic acids’ toxicity, with one indicating that “it is a work in progress, we are still trying to get CCME—Canadian Council of Ministers of the Environment—guidelines, and we are still trying to agree on a standard method to the chemistry” (INT-5). Despite researchers knowing from the beginning that they would work with no target or with a moving one, this absence of established toxicity values—values reflecting minimal damage to ecosystem components that regulators and the public find acceptable for release of the water—adds new layers of complexity: while this added complexity provided freedom, allowing scientists to perform foundational exploratory work, it also impacted the ability to gauge success rates for this project, thereby affecting budgeting, personnel, and overall researcher stress. Another interviewee furthered this observation by adding that “the challenge, with this project and with all oil sands related work, is that the wastewater that is produced by the oil sands is highly complex water chemistry that does not really boil down to one single value, one concentration or one chemical” (INT-8).

In sum, the micro-level discussions revealed that the genomics scientists involved in the project were facing obstacles in their local labs and environments related to cognitive complexity, like designing their programs, establishing novelty, and credibly sequencing their methodologies; considering the impact of their work on communities and regulators outside the laboratories; and managing their own stress as well as that of their staff and trainees.

### Macrosystem matters

Social and philosophical issues that emerged from the data connected individual scientists and the work in their laboratories to wider issues about scientific processes and accepted practices in different areas.

Attentiveness to a perceived “natural balance of things” in remediation emerged from researchers. One commented:

…oil is a product of millions of years of decomposition of plants through the use of microbes, so microbes are involved in sort of producing oil”, adding that “all the other things in the sand are also naturally present, like naphthenic acids… so, the balance is bringing the levels back to normal, because they just got concentrated through extraction (INT-2).

The idea implied here was that the project was remediating existing phenomena, which were an intensification of pre-existing phenomena. Another researcher elaborated on this theme with respect to microbes:

Sometimes it can be tricky because you know that they [microbes] would be doing a certain job, like degrading certain compounds, but they are still like living entities in the environment, so you don’t really know what the implications [of adding new organisms] are as well for the whole structure and functioning of the ecosystem. You don’t want to imbalance it and create a new problem down the road (INT-3).

These interests extended to how well scientists can mimic natural environments, convert bridge-controlled situations to natural-uncontrolled ones, and advance their research. One researcher, however, indicated that any equipment disturbs or perturbs a sample much like perturbations in the natural environment and that “the question will always be ‘what are we actually measuring? Does it make [it] back to real environment?’ And that often means we have to be very precise in our questions” (INT-5).

The question of applicability to the “real world” came up among other participants. They debated at what point the volume of laboratory work was enough to warrant application in real landscapes:

One of the things that we have to be very careful [about] as scientists is ensuring that we always understand that everything we do has limitations, has limited interpretations. … I guess there is kind of, like, one side where everything is natural, and the other side where you kind of artificially tease apart different parts of the system and then try to recreate it back. And that way is more scientifically controlled, but how representative is that of the real system? There are advantages and disadvantages of doing both of those things (INT-7).

A prominent topic among participants was how components of this program may be perceived by different social groups. For example, while one researcher was uneasy that microbial application would be well received by Indigenous communities, as they are perceived as having deeper respect for all elements of nature, another researcher was concerned if microbial application would be accepted at all, as these organisms might hold a “bad reputation” and people think of “their association with diseases” more than they consider their “environmental support” (INT-7). This range may be rooted in the fact that although microbial ethics and rights have been questioned before, little systemic attention has been given to the topic in this context, and there is still much to be debated about their biocentric values and uses, especially in a post-humanist approach (Cockell, 2011; Höll and Bossert, 2022). Participants also had concerns about the controversy related to genetic interventions (e.g., gene enhancement and gene modification) because it could limit the social license of genomics applications. One participant made this comment: “GE^3^LS should have another E, for education, as there is a lot going on; it is daunting, and people are afraid of words like genomics” (INT-2). However, studies have shown that it is not lack of knowledge that explains people’s resistance to technology-based advances, but rather other reasons like lack of trust, moral acceptability, and overconfidence (Gottweiss, 2002; Lewisch and Riefler, 2023; Fernbach et al., 2019). Hence, instead of perpetuating the idea of public deficits in knowledge (Cortassa, 2016), scientists should acknowledge peoples’ heterogeneity and invest in effective dialogue, making themselves more relatable and thereby enhancing trust (Seidel et al., 2023).

Additionally, participants described how the institution of academic publishing slows progress because it is oriented toward positive results and because researchers need “significant” findings before they can publish and be cited by others (Mlinarić et al., 2017; Bouchard and Larivée, 2022). The gaps in knowledge that would be filled by publishing negative results became a major theme with one participant asserting: “I always feel like somebody must have done this before, but when you read a paper, they make it sound so easy. It is like, why can’t I replicate this? This is not working for me” (INT-7). Another researcher discussed being on the editorial board of a journal specifically targeting negative findings. The participant said that the journal lasted less than 2 years before it was archived due to lack of submissions. The loss of this journal and the spotlight it cast on the importance of negative results is unfortunate, as it means many researchers may be making the same mistakes in their laboratories and slowing their progress through the lost opportunity to learn from others. As another interviewee indicated, “[negative results] happen all the time, we just spin the story differently and we still get publications”, adding “when we get unexpected results, those results are actually more interesting” (INT-5). These observations prompted us to ask the scientists who they thought they were publishing articles for. They stated that if other scientists would benefit from learning about unexpected results, there needed to be official outlets to share these results, incentivized through collegial processes or other means to relay these findings without judgement. Instead, they believed journals do not incentivize this work and sometimes even prevent these findings from being published, and scientific peers did not give adequate attention to the effort and ethic demonstrated by publishing negative results:

…if you are applying for a grant, that paper doesn’t really matter, and you are so busy that who has time to write a paper on negative data that doesn’t really contribute … There should be forums, not even peer-reviewed, where you could just throw raw data out there, just to summarize what you did and say ‘this didn’t work’, and so people don’t have to put a lot of effort into it, but can learn from others… (INT-4).

Even among this project’s interviewees, there were differences of opinion on the value of publishing negative results. Some considered a negative result a failure, while others embraced it as advancing the processes used for study. Some suggested using narrative devices to make a compelling story from negative results, while others said they just move on to the next experiment. One scientist revealed that they believed the existence of negative findings enhanced a study’s credibility and legitimacy and the study personnel’s overall humanity. This was surprising because not only does it refute other public perceptions of lab-coat scientists as overly ambitious and patronizing, but it also shows that researchers take their responsibilities seriously, reinforcing the idea of trust by relatability. Interestingly enough, here the team found ourselves in a conundrum: scientists are usually encouraged to make themselves invisible in their publications, letting only the data speak and consequently dehumanizing the research process, with visibility equating publication and citations (Seidel et al., 2023). However, this behaviour perpetuates distrust among the public because interpretations of results are necessarily tied to scientists’ positionality. In addition, publishing favours positive results because they achieve higher metrics, which could be seen as detrimental to quality dimensions voiced by other researchers, such as intellectual initiative and societal value (Aksnes et al., 2019; Helmer et al., 2020).

## Research-creation interpretation of findings

**FIGURE 3:**
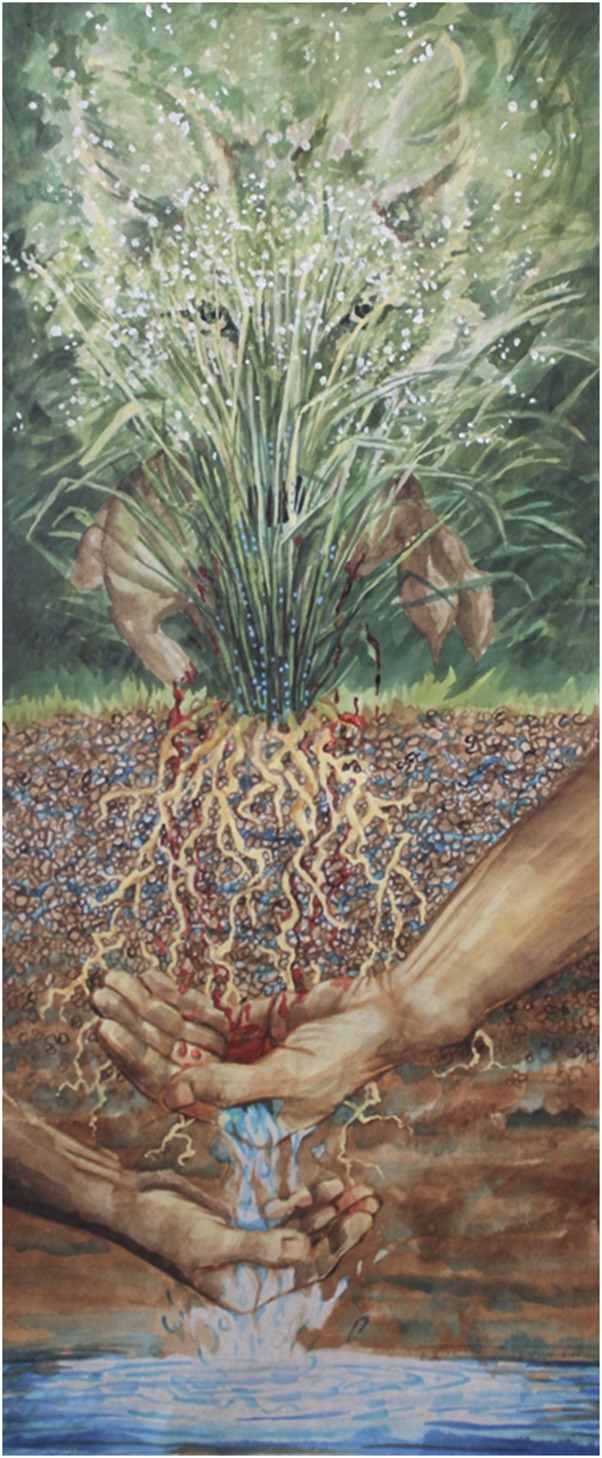
“Filtering” - Ink and acrylic on Mulberry paper, 18 w × 42 h resized.

As an extension to the project and a research-creation undertaking, the GE^3^LS project team included a visual artist who attended or reviewed recordings of all the virtual lab visits. The project team considered this individual an integral part of the research process, who would make art that could be more easily, accessibly, and diversely shared and interpreted than the research data and technical outputs. The goal was to inspire wider society to act on the messages that the art conveyed (Truman, 2023). According to the artist, the piece entitled “Filtering” (Figure 3),

symbolizes a constructed wetland within a “whole” ecosystem and the responsibility humans have to the whole. Plants and their associated microbes bestow on us a clean environment with their assistance, and they have these powers innately. The coyote represents the innate mystery of the natural world, and the tricks it will play are for us to learn from. If nature is to be “used” to work for humans, we invite the unknown and let go of control. Let’s bow, and honor natures innate balance, its mystery and powers, its all knowingness. Slender wheatgrass seeds are blowing in the sun, blood is running from the bunny, the bunny ate the grass, the coyote eats the bunny. We drink the water, our life source, filtered by plants and microbes synergistically. The blood falls onto the larger adult’s hand who holds the responsibility of ensuring safe, clean, water into the child’s hand and to future generations.

Hence, by symbolizing the interconnectedness of natural and constructed systems, perceptions and interpretations, the art fosters broader understanding of science and partnerships and highlights the ethical and societal implications of the research, making these insights relatable to diverse audiences.

## Conclusion

This work considered the role of researchers as more than just ‘science doers’, something not usually explored in the context of large-scale applied genomics projects. We used virtual visits and interviews to investigate the thoughts of lab-based genomics scientists and their teams situated in interdisciplinary genomics programs. While this study provides rich, context-specific insights into the micro- and macro-level dynamics of genomics research, it is exploratory in nature and not intended to generalize to all researchers in the field. One notable intersection between levels was the impact of academic publishing norms on the daily activities and morale of individual scientists. For instance, the systemic bias favouring the publication of positive results often led researchers to deprioritize reporting negative findings, which they acknowledged could significantly benefit their peers. This macro-level publishing pressure directly influenced micro-level laboratory practices, where researchers sometimes felt compelled to ‘spin’ their results or avoid investing time in projects unlikely to yield publishable data. Add to it the fact that the relationship between micro- and macro-matters is also self-reinforcing due to two-way interactions, representing a continuous challenge to the success of interdisciplinary approaches. Furthermore, a combination of how academic publishing potentially slows scientific progress with concerns on how genetic interventions could limit genomics’ social license, might lead people to believe that the absence of “flawed” studies could be indicative of science being manipulated. To mitigate those impressions and aware of the impacts of publishing norms on knowledge dissemination, we suggest genomics researchers to share all findings either in peer-reviewed or other repositories, such as institutional ones, science blogs, or as research briefs and comments in open-access venues.

Of notice was also the challenges of interdisciplinary collaboration, such as how scientists working together in the same research team and/or project may have different views on significant issues, and that these differences may affect group research. For example, participants asserted that using a particular technique may be a matter of experience, which leads to individual preferences, and, despite inputs from others, the final choice of approach and methods would rely on those performing the research. Therefore, we emphasize the importance of considering the social actors involved in a research project—researchers’ experience, background, connections, beliefs, and bias–affecting not only how the research is conceived and conducted, but also how the research data will be presented, received, and potentially put into action. We encourage agencies providing funding opportunities to account for resources for interdisciplinary team support, such as conflict resolution, knowledge translation between disciplines, and no-cost extensions to interdisciplinary work recognizing the time it takes to develop a shared language.

We recommend that social assessments like this be done at the beginning of every project, as they will allow the identification of features that may impact the research along the way, especially among divergent research domains like social science and genomic sciences, philosophers and lab-based scientists. We would also like to see these social assessments published, as although the literature on this topic is very limited, the technical, social, and philosophical questions that emerged here may be common to different research teams.

By shifting the spotlight from the research to the investigators as subjects, this study suggests that scientists are keenly aware of these and other problems, enabling the public to better relate to researchers, enhancing trust in the scientists, and making science more accessible. Also, the shift of scientists into subjects of an artist’s interpretation, allows these professionals to learn about the ways they are perceived by others, and in turn, to reflect on their own activities. In addition, the translation of results into artwork, such as in “*Filtering*”, deepens engagement and appreciation of multiple knowledge systems by communicating complex scientific themes through accessible visual narratives.

One limitation of this study is its small sample size, as it involved only eight genomics researchers from a single interdisciplinary project. As a result, the findings may not be fully generalizable to other genomics research teams or projects. Additionally, the exploratory case study and grounded approach focuses on in-depth, context-specific insights, which may not capture the broader trends across the genomics field. We did not seek to confirm hypotheses in the work, but to begin discussion on positionality of genomics scientists within their respective projects. Finally, the virtual nature of the laboratory visits may have further limited the observational depth compared to in-person interactions. Additional work could include large-scaled surveys of genomics scientists, focus groups, more case studies such as this one, and increased dialogue in publication venues to build a comprehensive understanding of the philosophies and social dynamics of genomics scientists in interdisciplinary programs, which can help inform the sociology of science and public engagement with genomic sciences.

By framing our findings as a case study, we aim to open avenues for broader discussions on the interplay between individual experiences and systemic challenges within interdisciplinary projects. Addressing such systemic barriers is critical to fostering a more open and supportive research environment. In conclusion, future research should prioritize frameworks that support interdisciplinary teams by fostering mutual understanding and streamlining collaboration; policy reforms could include incentivizing the publication of methodological challenges and negative results to enhance transparency and learning; and public engagement strategies should aim to demystify genomics, emphasizing its societal relevance and addressing public concerns about genetic interventions.

## Data Availability

The raw data supporting the conclusions of this article will be made available by the authors, without undue reservation.
